# Antifungal Carvacrol Loaded Chitosan Nanoparticles

**DOI:** 10.3390/antibiotics11010011

**Published:** 2021-12-22

**Authors:** Alberto Vitali, Annarita Stringaro, Marisa Colone, Alexandra Muntiu, Letizia Angiolella

**Affiliations:** 1 Istituto di Scienze e Tecnologie Chimiche “Giulio Natta”, Consiglio Nazionale delle Ricerche, L. go F. Vito 1, 00168 Rome, Italy; alexandra.muntiu@gmail.com; 2 National Center for Drug Research and Evaluation, Italian National Institute of Health, V. le Regina Elena, 299, 00161 Rome, Italy; annarita.stringaro@iss.it (A.S.); marisa.colone@iss.it (M.C.); 3 Department of Public Health and Infectious Diseases, “Sapienza” University of Rome, P. le Aldo Moro, 5, 00185 Rome, Italy; letizia.angiolella@uniroma1.it

**Keywords:** carvacrol, chitosan, nanoparticles, *Candida*, antifungal, biofilm

## Abstract

The increased prevalence and incidence of fungal infections, of which *Candida albicans* represents one of the most life-threatening organisms, is prompting the scientific community to develop novel antifungal molecules. Many essential oils components are attracting attention for their interesting antifungal activities. Given the chemical and physical characteristics of these compounds, the use of appropriate nanodelivery systems is becoming increasingly widespread. In this study, chitosan nanoparticles were prepared using an ionic gelation procedure and loaded with the phenolic monoterpene carvacrol. After a bioassay guided optimization, the best nanoparticle formulation was structurally characterized by means of different spectroscopic (UV, FTIR and DLS) and microscopy techniques (SEM) and described for their functional features (encapsulation efficiency, loading capacity and release kinetics). The antifungal activity of this formulation was assayed with different *Candida* spp., both in planktonic and biofilm forms. From these studies, it emerged that the carvacrol loaded nanoparticles were particularly active against planktonic forms and that the antibiofilm activity was highly dependent on the species tested, with the *C. tropicalis* and *C. krusei* strains resulting as the most susceptible.

## 1. Introduction

Essential oils (EO) have widespread uses in the health, agricultural, food, cosmetic and pharmaceutical industries due to their potent antimicrobial activities. Many single components of the EO maintain the antimicrobial potency of the whole oil mixture. Various examples are also reported in which EOs single components were delivered in different nano-systems [1]. Carvacrol (Cv), a monoterpene phenol and one of the main components of oregano (*Origanum vulgare*) EO [2], features many interesting biological properties [3]. Known as a food preservative, it also possesses potent antioxidant activities comparable to those of ascorbic acid and vitamin E [4]. Carvacrol is a potent antimicrobial agent against molds and Gram-positive and Gram-negative bacteria. Its antimicrobial activities were tested in different studies [5]. In particular, its antifungal action was evaluated against human pathogens such as *Candida* spp. [6,7] and *Malassezia* spp. [8,9] showing remarkable efficacy. In a recent study, carvacrol treatment was shown to cause permeability and depolarization of the cell membrane of *Candida albicans* cells. In addition, after carvacrol treatment, apoptosis-related markers (DNA fragmentation and metacaspase activation) were observed, and total and mitochondrial reactive oxygen species (ROS) levels were increased, as long as the cytosolic and mitochondrial calcium levels did. Furthermore, carvacrol was shown to have low toxicity and to be effective in alleviating systemic *C. albicans* infections through its antifungal and immunomodulatory activities [10]. The chemical structure of carvacrol makes this compound a volatile molecule at room temperature, with an almost complete lack of idrosolubility. Furthermore, carvacrol at high concentrations may be toxic to many cells [11]. Thus, to enhance carvacrol bioavailability and stability, while at the same time limiting potential negative side-effects, different nano-formulations have been proposed for its delivery to be applied as food preservative [12,13,14] or to contrast bacterial strains [15,16]. Chitosan (Cs), a polymer obtained for deacetylation of the natural compound chitin, is greatly consolidated in nano- and micro-delivery applications, due to its characteristics of biocompatibility, biodegradability, low toxicity, and high hydrophilicity [17,18], and because of the relative ease in preparing nano or microparticles [19,20]. Moreover, it can be used to prepare films, membranes, gels, beads, fibers [21,22]. Cs is widely used in the preparation of antimicrobial nanodevices for its intrinsic antibacterial properties against Gram-positive and Gram-negative bacteria and towards fungal pathogens [23,24]. Different kinds of molecules (small molecules, peptides, nucleic acids) may be administered through mucous membranes thanks to the ability of Cs to stimulate transient opening of the tight junctions of the cell membrane. It is available in different size preparations from low to high molecular weights, allowing a fine-tuning of the nano- or micro-particles dimensions. High molecular weight chitosans are generally less soluble in water; conversely, low molecular wight Cs are more hydrosoluble and easier to manipulate for the production of nanoparticles. Additionally, the presence of free amino groups renders the Cs nanodevice prone to be functionalized with other molecules, for example antimicrobial peptides [25] cell-penetrating peptides (CPPs) [26] or tumor homing peptides [27] for a targeted and highly specific delivery of the desired drugs.

*Candida* spp. are among the most frequent nosocomial pathogens, contributing significantly to morbidity and mortality of hospitalized patients. *Candida albicans* was the most prevalent species (50.7%), followed by *Candida parapsilosis* (17.4%), *Candida glabrata* (16.7%) and *Candida tropicalis* (10.2%). The prevalence of non-albicans Candida spp. increased over time [28]. A major virulence attribute of *Candida albicans* is in fact related to its ability to form biofilms, densely packed communities of cells intrinsically resistant to conventional antifungal therapeutics, the host immune system, and other environmental factors, making biofilm-associated infections a significant clinical challenge [29]. Biofilm formation, although being a process present in all the Candida species focused above, differs significantly from species to species, and in the dependency of surface, host niche and other factors. Biofilm characteristics depend on the ability of each species to produce extracellular polymeric substances (EPS) and display dimorphic growth, but also on the biofilm substratum, carbon source availability and other factors [30].

In this study we have explored the possibility to couple the antifungal properties of Cs and Cv, preparing nanoparticles based on low molecular weight Cs and testing their efficiency towards different strains of *Candida*. After a bioassay guided optimization for the preparation of efficient Cv loaded nanoparticles, the final formulation composed of a Cs/Cv 1:1.5 (*w*/*w*) ratio, was characterized by UV spectrophotometry, attenuated total reflectance-Fourier transform infrared spectroscopy (ATR-FTIR), scanning electron microscopy (SEM), dynamic light scattering (DLS). Encapsulation efficiency (EE) and loading capacity (LC) were also evaluated as long with the release kinetic. To evaluate the antifungal properties of the so prepared carvacrol-loaded chitosan nanoparticles, they were challenged with *Candida albicans*, *Candida krusei*, *Candida glabrata* and *Candida tropicalis* strains both in planktonic and biofilm forms.

MIC values, killing kinetics and effects on pre-formed and established biofilm have been also evaluated.

## 2. Results and Discussion

### 2.1. NPs Preparation

Empty chitosan nanoparticles (Cs-NPs) loaded with carvacrol (Cv–Cs-NPs), were prepared as reported in the Materials and Methods (Section 3) following an ionotropic gelation-based procedure. In order to determine the formation of carvacrol-loaded chitosan nanoparticles the Cv loaded nanoparticles obtained with different Cs/Cv ratios were analyzed firstly by UV-VIS spectrophotometry.

The maximum absorption peak of the carvacrol–ethanol solution is at 275 nm, as reported elsewhere [31]. The supernatant of chitosan particles immersed in ethanol for 1 h showed no absorption peak at wavelengths between 240 and 350 nm. On the other hand, the obtained solution containing loaded NPs after centrifugation showed a maximum absorption peak at 275 nm, clearly indicating the presence of carvacrol and consequently, its loading into chitosan particles.

### 2.2. Bioassay Guided Nanoparticles Optimization

In a first set of the experiments aimed to optimize the antifungal effect of different Cs/Cv formulations, we assayed the antimicrobial activity of chitosan nanoparticles loaded with different amounts of carvacrol against *C. albicans* AIDS 68 strain taken as reference. In Table 1 are reported the obtained results. The data shown that the MICs were about >2400 µg/mL for all the nanoparticles characterized by a *w*/*w* ratio in favor of Cs. NPs produced with a 1:1 (Cs/Cv, *w*/*w*) ratio showed an antifungal activity identical to that of free Cv (MIC 1200 µg/mL). An enhancement in the antifungal activity could be achieved augmenting the Cs/Cv in favor of Cv, with a dramatic effect obtained when carvacrol was loaded in a ratio of 1:1.50 (Cs/Cv, *w*/*w*), with a resulting MIC of 24 µg/mL (Table 2). Interestingly, this value resulted highly lower respect free Cv indicating a good loading efficiency of this formulation. In particular, a dramatic difference could be observed between the 1:1.25 and 1:1.5 ratios. Such a difference in the effect given by the two formulations is difficult to interpret. As observed in many biological systems such an effect is probably due to the complexity in the response of the biological system itself to loaded nanoparticles as reported by Bell and coll. [32].

Consequently, this formulation, was used for all the following experiments to test the antimicrobial and antibiofilm activity against different *Candida* species.

### 2.3. Cs–Cv-NPs Characterization

**Figure 1:**
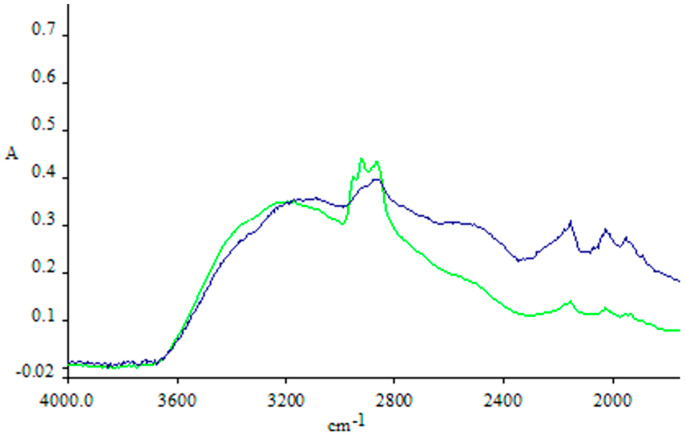
ATR-FTIR spectra of Cs-NPs Chitosan (blue line) and Cv–Cs-NPs (green line).

In order to characterize the Cv–Cs-NPs obtained from the optimization rounds, different analytical techniques have been employed. After the UV analysis to confirm the presence of CV, a further characterization of Cv–Cs-NPs was made by means of ATR- FT-IR analysis. Although the spectra of the products obtained with the addition of carvacrol are similar to that of the chitosan particles, the intensity of the CH stretching peak at 2870–2960 cm^−1^ increases significantly, indicating the presence of carvacrol in the chitosan matrix, as reported in Figure 1. Similar signals have been obtained in other studies relating to carvacrol encapsulation in chitosan nanoparticles [33] confirming our data on the successful incorporation of the monoterpene in Cv–Cs-NPs.

**Figure 2:**
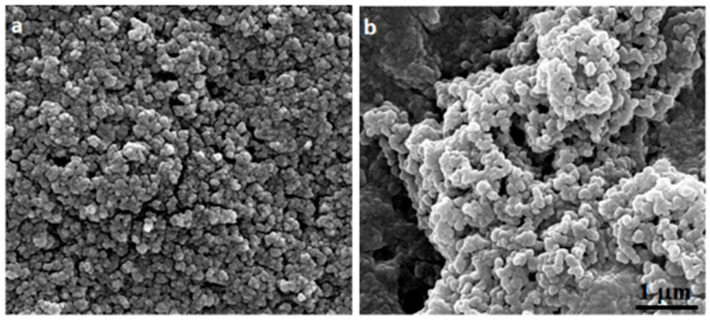
SEM images of empty chitosan NPs (**a**) and carvacrol-loaded nanoparticles (**b**).

The morphology of empty chitosan Cs-NPs and Cv–Cs-NPs were observed by scanning electron microscopy (SEM), with both showing a spherical shape. As is evident from the image (Figure 2a,b), the nanoparticles are in a high aggregated state due to the air dehydrated environment used to prepare SEM samples. This is also not surprising given their size in the range of 150–400 nm and the fact that the preparation process chosen was O/A emulsion.

Furthermore, similar morphology and aggregation patterns were observed in different studies concerning the preparation of chitosan nanoparticles [34,35].

The average dimensions in terms of hydrodynamic radius of the particles, was investigated by means of dynamic light scattering (DLS) technique. The average dimensions of obtained Cs-NPs and Cv–Cs-NPs were above the canonical 100 nm to be considered nanoparticles, however in the biomedical field the size range of NPs was extended to 1000 nm. The empty chitosan nanoparticles and the loaded carvacrol nanoparticles showed a hydrodynamic diameter of 384.5 and 281.6 nm respectively (Table 2) in accordance with other studies involving the encapsulation of Cv [32] and other phenolic compounds characterized by similar dimensions of carvacrol [36,37,38]. The empty chitosan particles and the Cv–Cs-NPs yielded similar PDI values of 0.235 and 0.2475 respectively (Table 1) indicating an acceptable grade of monodispersity [39].

The encapsulation efficiency (EE), loading capacity (LC) and the release kinetic of Cv were also evaluated. The nanoparticles obtained by mixing Cs and Cv in a 1:1.5 (*w*/*w*) showed percentages of 56% of EE and a 25.5% of LC calculated respectively using the Equations (1) and (2) as reported in the Materials and Methods (Section 3).

The timing of carvacrol release from nanoparticles was observed using a buffer solution at pH 7. The study was carried out for 5 days, taking samples at different incubation time checkpoints (0, 24, 48, 72, and 96 h). The amount of carvacrol released in the supernatant was calculated by reading the UV absorption at 275 nm.

**Figure 3:**
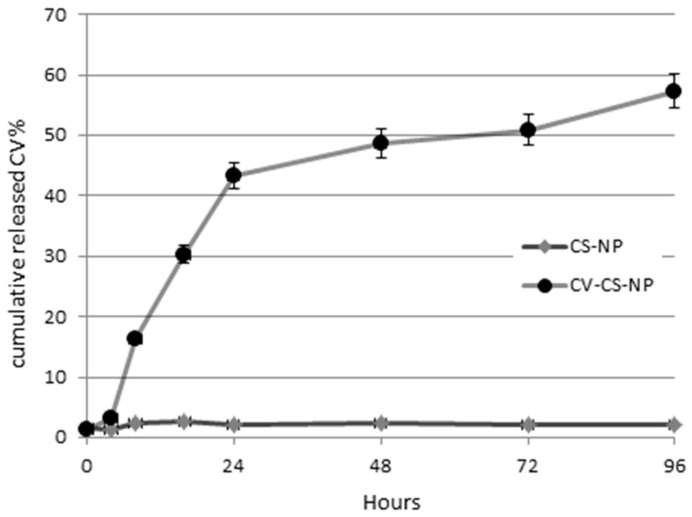
Kinetic of cumulative Cv release at pH 7.4 at 37 °C in five days from Cv–Cs-NPs prepared with a Cs/Cv 1:1.5 (*w*/*w*) ratio. Results are reported as mean ± SD, n = 3.

The release of carvacrol from the chitosan–carvacrol particles at 37 °C was followed for five days and resulted to be very fast in the first two days when 42% of cumulative release of Cv was calculated, after which a slow release could be observed (Figure 3). Similar behavior was observed for released chlorogenic acid [38] and ciprofloxacin [40] chitosan nanoparticles in similar pH conditions.

### 2.4. Evaluation of Antimicrobial Activity

In all the following experiments freshly prepared Cs-NP and Cv–Cs-NPs were prepared to avoid loss of efficacy. The antimicrobial activity of Cv–Cs-NPs was tested on four *Candida* spp., specifically *C. albicans* AIDS 68, *C. glabrata* SFY115, *C. tropicalis* 47829 and *C. krusei* 45709 (Table 3).

The MIC values for free Cv are the same for all the strains assayed, while a marked difference was shown for Cv–Cs-NPs. In this case the lowest MIC value (24 µg/mL) was obtained only in *C. albicans*, while for the other strains it ranged from 780 µg/mL to 1560 µg/mL. These results may account for a specie-specific dependent mode of action of these NPs. Similar results were reported by other authors with green synthesis of silver nanoparticles [41]. On the other side, free Cs and Cs-NPs showed MIC values generally higher respect Cv and Cv–Cs-NPs, although a species dependent mode of action could be observed.

**Figure 4:**
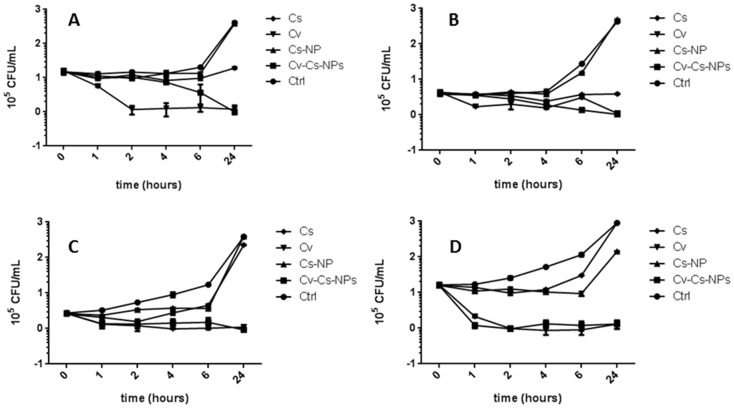
Time-killing curves of control (Ctrl), carvacrol loaded NPs (Cv–Cs-NPs), empty NPs (Cs-NPs), chitosan (Cs) and free carvacrol (Cv), against *C. albicans* (**A**), *C. glabrata* (**B**), *C. krusei* (**C**) and *C. tropicalis* (**D**) at different interval time. Values are reported as mean ± SEM, n = 3.

To assess the kinetics of the killing effect of Cv–Cs-NPs, time-killing experiments were carried out on *C. albicans*, *C. glabrata*, *C. krusei* and *C. tropicalis* strains with Cv–Cs-NPs, Cs-NPs, Cv, Cs alone at the respective MIC concentrations (Figure 4A–D).

In all the cases free Cs and Cs-NPs were not able to kill the cells, at least the effect of free Cs was similar to the control for *C. krusei* (Figure 4C) and for *C. tropicalis* (Figure 4D) or resulted in a reduced growth for *C. albicans* (Figure 4A) and for C. glabrata (Figure 4B).

Conversely, in all the species considered, Cv–Cs-NPs and free Cv, were able to kill the cells, but with different rates. In *C. albicans* (Figure 4A) Cv–Cs-NPs were able to inhibit cell viability after 6 h while free Cv exerted the same effect after 2 h. In *C. glabrata* (Figure 4B) the killing effect of Cv–Cs-NPs is only evident after 24 h of incubation, the same time required for free Cv too. In *C. krusei* (Figure 4C) and in *C. tropicalis* (Figure 4D) the Cv–Cs-NPs were able to kill the cells after 2 h and 1 h respectively, similarly to free Cv. These results show that the effects of free or loaded Cv are almost superimposable such in the case of *C. glabrata*, *C. krusei* and *C. tropicalis*, while only for *C. albicans* a difference in the time killing between free Cv and cargoed Cv is evident, indicating a gradual release of the monoterpene from NPs.

The differences observed in killing efficacy and kinetics, may strongly depend on the different distribution of polysaccharides within the cell wall of each *Candida* species as observed after treatments with different synthetic antifungal drugs [42,43,44], influencing both the interaction of chitosan NPs with the different cell wall architectures and the subsequent effects of the substances on the cells.

### 2.5. Inhibition on Pre-Formed and Forming Biofilm by Cv–Cs-NPs in Candida spp.

**Figure 5:**
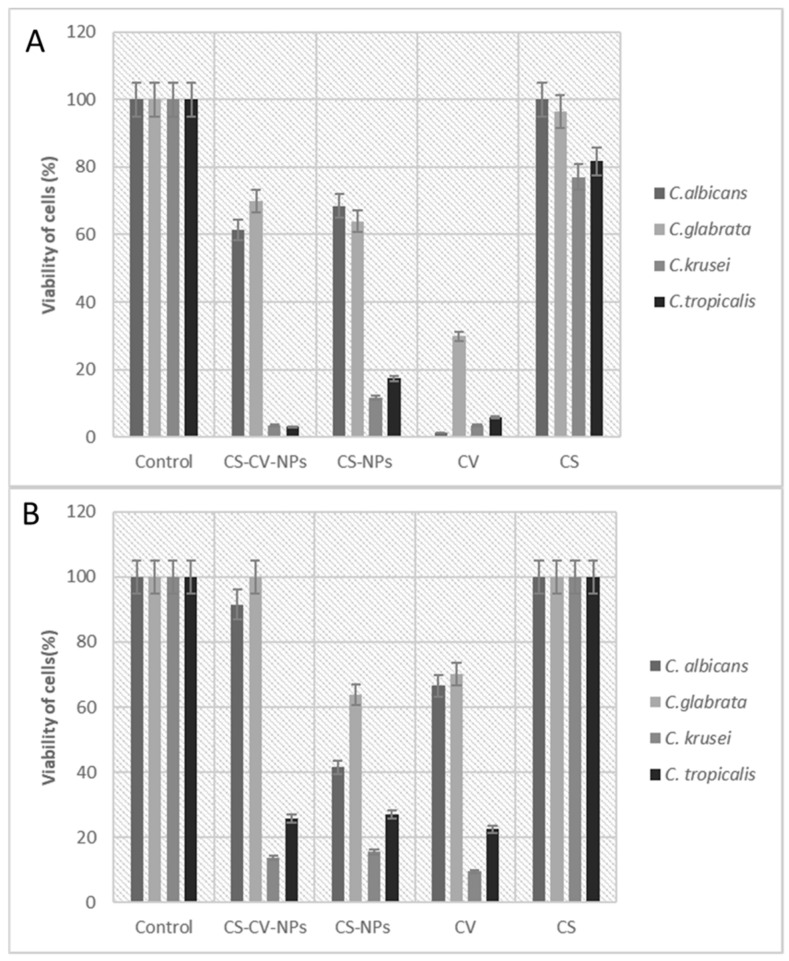
Cell viability expressed as % of XTT reaction product read at 490 nm. (**A**) NPs added at beginning of biofilm formation, (**B**) NPs added on preformed biofilm. Treated cells were compared to the control (one-way ANOVA followed by Dunnett’s post hoc test, *p* < 0.05).

To verify the possible inhibitor effect of Cv–Cs-NPs on biofilm produced by different *Candida* species, we carried out XTT assays at beginning of biofilm formation and on pre-formed biofilms. Figure 5 reports the viability in presence of Cv–Cs-NPs, Cs-NPs, Cs and free Cv, at beginning of biofilm formation (A) and on pre-formed biofilm (B). As a result, Cv–Cs-NPs were able to inhibit biofilm formation at 30% and 40% in *C. glabrata* and in *C. albicans* respectively (Figure 5A), while in *C. krusei* and *C. tropicalis* (Figure 5A) inhibition on forming biofilm was around 90%. It is surprisingly that the effect of Cs-NPs was almost superimposable to that due to Cv–Cs-NPs, with only small differences in the case of *C. krusei* and *C. tropicalis*.

On the other hand, free Cv differently inhibited forming and pre-forming biofilm depending on the strain considered. In preformed biofilm, it decreased the viability of about 70% in *C. glabrata*, while on *C. albicans*, *C. krusei* and *C. tropicalis* inhibition reached the 90%. Similarly, on forming biofilm, Cv was more effective against *C. krusei* (90%) and *C. tropicalis* (70%), but in a lesser extent towards *C. albicans* and *C. glabrata* (around 30% of inhibition).

Finally, Cs alone slightly inhibited biofilm formation in *C. krusei* and *C. tropicalis* (20% of effect), but no effect was visible on the other two species (Figure 5A); in the same manner no effect was detectable on preformed biofilm in all the strains (Figure 5B). It is also interesting to note that Cs alone in form of not aggregated polymer and in form of nanoparticle, behave differently. Cs-NPs despite the absence of Cv, are effective in a similar manner to Cv–Cs-NPs and to free Cv. This aspect may be explained considering that Cs is known to have an intrinsic antibiofilm activity [45,46,47], but the nanostructure of the NPs may enhance this feature being more effective in penetrating the complex biofilm network [48].

These results firstly suggest that Cv–Cs-NPs showed to be globally more efficient on *C. tropicalis* and *C. krusei* both on pre-formed and forming biofilms. But a similar effect is also due to unloaded Cs-NPs and to free Cv indicating that the encapsulation does not enhance Cv efficiency and that the effect is almost unspecific and not due to a synergistic or additive effect between Cs and Cv.

Moreover, these data suggest that differences in biofilm structures among the considered species, may affect the response to NPs treatment observing that *C. krusei* and *C. tropicalis* biofilm seem more susceptible either in a pre-formed or in formation state. These differences may be dependent on the type of biofilm formation and structure.

It was observed that *C. albicans* mature biofilms exhibit a more heterogenous structure, composed by blastophores and hyphae surrounded by an extracellular material (ECM) of polysaccharide material [49]. In the case of *C. glabrata*, the biofilm is exclusively composed by yeast cells forming a multilayer and intimately packed structure or arranged in cell clusters [50]. In turn, *C. tropicalis* biofilm corresponds to a network of yeast, pseudohyphae and hyphae, with intense hyphal budding [51].

## 3. Materials and Methods

### 3.1. Chemicals

Na-Tripolyphosphate and chitosan (Cs) at ultra-low molecular weight Mw of 20 kDa and a deacetylation degree of 95% (Glentham Life Sciences, Corsham, UK) were used in this study. Carvacrol (density 0.98 g/mL and PM 150.22 g/mol) and all other chemicals (ethanol, acetic acid, XTT, menadione) unless specified, were purchased from Sigma-Aldrich (Milan, Italy).

### 3.2. Nanoparticles Preparation

Chitosan–carvacrol nanoparticles were prepared as reported [33]. A 1.2 % *w*/*v* chitosan solution was prepared by dissolving 1.2 g of chitosan in 100 mL of a 1% *v*/*v* aqueous acetic acid solution while stirring at room temperature overnight. The following day, Tween 60 (0.7 mL) was added to the chitosan solution and left to warm at 60° for 2 h under continuous stirring to obtain a homogeneous solution. At the end of 2 h, carvacrol was slowly added to the solution, leaving it to stir for 20 min. In optimization experiments, increasing amounts of carvacrol, i.e., 0.12, 0.24, 0.36, 0.48, 0.60 and 0.72 g were used to obtain different weight ratios of chitosan to Cv of 1:0.25; 1:0.50; 1:0.75; 1:1.0; 1:1.25; 1:1.50 and 0.25:1 respectively. The solutions were divided into several aliquots, and carvacrol was added proportionally to the total volume. Next, a solution of TPP (0.5% *w*/*v* 40 mL) was slowly added to the O/A emulsion that was forming and left to stir for 30 min. The final pH of the solution was 5.0. Finally, the particles were obtained by centrifugation at 10,000 rpm for 10 min, after which the supernatant was removed from each sample and the resulting pellet washed with an aqueous solution of Tween 60 (1% *v*/*v*) to remove free carvacrol. The same procedure was replicated to obtain carvacrol-free nanoparticles used as controls.

### 3.3. Nanoparticles Characterization

#### 3.3.1. Scanning Electron Microscopy (SEM)

SEM analysis allowed the study of chitosan nanoparticle morphology. Samples were deposited on 12 mm-diameter glass coverslips for 30 min and then were gold coated by sputtering (SCD 040 Balzers device, Bal-Tec). The samples were then examined with a scanning electron microscope FEI Quanta Inspect FEG, (FEI, Hillsboro, OR, USA) at 30 kV.

#### 3.3.2. Attenuated Total Reflectance Fourier-Transform Infrared Spectroscopy (ATR-FTIR)

Spectra were acquired with a Spectrum One spectrophotometer (Perkin-Elmer, Milan, Italy) equipped with an ATR accessory having a ZnSe reflection element. The samples were solubilized in water by placing 3 µL of 1 mg/mL solution on the crystal and left to air dry. Before sample analysis, an open beam background spectrum of a clean crystal was recorded; the spectra were acquired in Absorbance, between 4000 and 700 cm^−1^ after 40 scans at a resolution of 1 cm^−1^. During the acquisitions at 25 °C, the crystal was continuously placed under nitrogen flow to remove residual water.

#### 3.3.3. Dynamic Light Scattering

Nanoparticle size distribution and ζ potentials were obtained by DLS analysis performed with a Zetasizer Nano-S instrument (Malvern Instruments, Malvern, UK). Samples were dispersed in water obtaining a concentration of 1 mg/mL and centrifuged at 4000 rpm for 5 min at 10 °C. The hydrodynamic diameter distribution of the samples was measured using protein refractive index (1.63) and water refractive index (1.33) as working parameters for the samples and solvent, respectively. Size results were reported as the Z average of the particle hydrodynamic diameter distribution of three measurements at 25 °C of at least 10 runs repeated three times.

#### 3.3.4. Evaluation of Encapsulation Efficiency and Loading Capacity

Nanoparticle’s preparations (200 µL) after an ultrafiltration step employing 30 kDa MW cut off filters to eliminate free carvacrol, were mixed with aqueous hydrochloric acid solution (2 N) for a final volume of 2 mL and boiled at 95 °C for 30 min. After cooling down, ethanol (1 mL) was subsequently added to the mixture. A blank solution was similarly prepared replacing the sample dispersion with ultrapure water. After centrifugation at 10,000 rpm for 5 min at 25 °C, the supernatant was collected and analyzed by a spectrophotometer at wavelengths ranging from 278 nm. The encapsulation efficiency (EE) and loading capacity (LC) of carvacrol were calculated from Equations (1) and (2), respectively:

%EE = mass of loaded carvacrol/mass of initial carvacrol × 100(1)

%LC = mass of loaded carvacrol/mass of sample × 100(2)

### 3.4. Release Kinetics

The release kinetics of Cv from the chitosan particles were analyzed in phosphate buffered saline (PBS), the same used for the biological assays. The pellet of free and loaded nanoparticles was resuspended in 0.8 mL of PBS. The mixture was then left in gentle agitation for the required time at 25 °C. At different intervals of incubation time (0 h, 24 h, 48 h, 72 h, and 96 h), 100 µL of the sample was taken, centrifuged at 9000 rpm for 5 min at 25 °C. An equal volume of PBS was then added to the solution to restore the original volume. The amount of carvacrol released into the supernatant was calculated by UV spectrophotometer considering the maximum absorption wavelength of carvacrol (278 nm). The spectrum and concentrations of carvacrol were determined by UV-Vis Spectrophotometer (8453 UV-Vis Supplies, Agilent Technologies, Santa Clara, CA, USA) by diluting the compound in ethanol. The calculated molar extinction coefficient for carvacrol in ethanol was 2.650 M^−1^ cm^−1^.

### 3.5. Organisms and Growth Conditions

*Candida* spp. strains from clinical isolates were used through the study. In particular, the strains were *C. albicans* AIDS 68, *C. glabrata* SFY115, *C. tropicalis* 47829 and *C. krusei* 45709. Each strain was routinely maintained on Sabouraud dextrose agar medium (SDA; Difco, Detroit, MI, USA) at 28 °C.

### 3.6. Evaluation of Antimicrobial Activity

The antimicrobial activity of the chitosan–carvacrol nanoparticles (Cv–Cs-NPs), chitosan nanoparticles (Cs-NPs), chitosan (Cs) and carvacrol (Cv) were evaluated by a microbroth dilution method according to Clinical and Laboratory Standards Institute (CLSI), Approved Standard M27-A3, 2008. The minimal inhibitory concentration (MIC) was determined as the lowest concentration of Cv–Cs-NPs, Cs-NPs, Cs or Cv at which no microbial growth was observed, and were tested against four clinical isolates of Candida spp. Cv–Cs-NPs, Cs-NPs, Cs and Cv were diluted in RPMI 1640 supplemented with MOPS and Tween 20 (final concentration of 0.01% *v*/*v*). The dilutions ranging from 0.006 to 1.56 mg/mL. The inoculum size was about 2.5 × 10^3^ cells/mL. The plates were incubated at 28 °C for 24–48 h.

### 3.7. In Vitro Time–Kill Kinetics

Time–kill curve methods were used to evaluate the activities of Cv–Cs-NPs, Cs-NPs, Cs or Cv against different *Candida* spp. An overnight broth culture of *Candida* spp. was diluted in Sabouraud broth to obtain a starting inoculum of about 1 × 10^5^ CFU/mL. Substances were added to the broth culture at concentrations of MIC. 100 μL aliquots of broth were taken after 0, 30, 60, 120, 360 min and 24 h of incubation. Each aliquot was serially diluted, plated onto Sabouraud agar plates in duplicates and incubated at 28 °C to 48 h. The number of colony forming units was counted and CFU/mL was determined. The values were plotted to obtain the kinetics of killing. Results are expressed as CFU/mL.

### 3.8. Biofilm Formation

Biofilm formation was tested in presterilized polystyrene flat-bottom 96-well microtiter plates (Corning, Corning, NY, USA). A *Candida* spp. cell suspension (1 × 10^6^ cells/mL), at grown, was incubated for 48 h at 37 °C. After 3 h or 24 h of biofilm formation, Cv–Cs-NPs, Cs-NPs, Cs or Cv at their respective MIC concentrations, were added. After 24 h a semi quantitative measurement of biofilm formation was made by using an XTT[2,3-bis(2-methoxy-4-nitro-5-sulfo-phenyl)-2H-tetra-zolium-5-carboxanilide]-reduction assay [52] XTT was dissolved in PBS at 0.5 g/L. The solution was sterilized through a 0.22 µm pore size filter. Prior to each assay, the XTT solution was thawed and supplemented with menadione (10 mM stock dissolved in acetone to a final concentration 1 µM). An aliquot of 1 mL of the XTT-menadione solution was added per well, and the plates were incubated for 2 hat 37 °C. A sample (500 µL) was then transferred from each well into a fresh 12-well plate (to eliminate interference of cells with colorimetric readings) and the colorimetric change, resulting from XTT reduction, was measured at 490 nm.

### 3.9. Statistical Analysis

Statistical analysis was performed by GraphPad Prism (version 5.0, GraphPad Software Inc., San Diego, CA, USA) by using one way ANOVA followed by Dunnett’s post hoc test. (*p* < 0.05).

## 4. Conclusions

In this study, chitosan nanoparticles produced by means of an ionic gelation method were loaded with the phenolic monoterpene carvacrol. From a bioassay guided optimization, the best formulation of 1:1.5 Cs/Cv (*w*/*w*) was characterized by means of FTIR, UV spectroscopy and DLS, and tested against four different clinical isolated strains of *Candida* spp. represented among the most known pathogenic species: *C. albicans* AIDS 68, *C. glabrata* SFY115, *C. tropicalis* 47829 and *C. krusei* 45709. The antimicrobial activity, the time killing, and the inhibition of cells organized in the biofilm at the initial stage and as an established form have been evaluated.

The Cv–Cs-NPs showed to possess a better antifungal effect compared to free Cv being more effective against *C. albicans* strain with a MIC of 24 µg/mL, and were also able to affect viability of cells in the pre-formed and forming biofilm, although in this case, similar effects could be also observed upon treatment with Cs-NPs and free Cv.

An evident species-specific relationship could be observed regarding the effect of free and loaded Cs-NPs, highlighting the different structural organization existing among the tested species and between the planktonic and biofilm forms suggesting that at least in the case of *Candida*, a more refined optimization of the production of antibiofilm Cv–Cs-NPs should be carried out in relation to the target species.

## Figures and Tables

**Table 1:** Antimicrobial activities of NPs obtained with different Cs/Cv loaded in Cs-NPs *C. albicans* against AIDS 68.

Material	Cs/Cv (*w*/*w*)	MIC µg/mL
Cs	-	>2400
Cv	-	1200
Cs-NPs	-	1580
Cv–Cs NPs	1:0.25	>2400
Cv–Cs-NPs	1:0.50	>2400
Cv–Cs-NPs	1:0.75	>2400
Cv–Cs-NPs	1:1	>2400
Cv–Cs-NPs	1:1.25	1200
Cv–Cs-NPs	1:1.50	24

**Table 2:** Size and surface charge parameters of Cs-NPs and Cv–Cs-NPs in PBS buffer. Values are reported as mean ± SEM, n = 3.

	Cv–Cs-NP	Cs-NP
Hydrodynamic diameter (nm)	281.6 ± 2	384.5 ± 3
Polydispersity index (PdI)	0.235 ± 0.03	0.2475 ± 0.02

**Table 3:** MIC Antimicrobial activity of chitosan–carvacrol nanoparticles (CV–CS NPs) against *Candida* spp. The concentrations of the different compounds are expressed as µg/mL.

Strains	Cs	Cv	Cs-NPs	Cv–Cs-NPs 1:1.50
*C. albicans AIDS68*	1560	780	1560	24
*C. tropicalis 47829*	>3120	780	3120	1560
*C. krusei 45709*	1560	780	3120	1560
*C. glabrata SFY115*	1560	780	1560	780

## Data Availability

Not applicable.
